# Acute Demyelination in a Person with Amphetamine Abuse

**DOI:** 10.1155/2011/514613

**Published:** 2011-10-18

**Authors:** Serge Weis, Hans Sachs, Andreas Büttner

**Affiliations:** ^1^Laboratory of Neuropathology, Department of Pathology and Neuropathology, State Neuropsychiatric Hospital Wagner-Jauregg, Wagner-Jauregg-Weg 15, 4020 Linz, Austria; ^2^Institute of Legal Medicine, University of Rostock, 18055 Rostock, Germany

## Abstract

We report the case of a 31-year-old woman, admitted to the hospital for chest pain, dying a few days later from septic multiorgan failure, and showing at autopsy foci of acute demyelination in the occipital lobe. Gas chromatography/mass spectrometry analysis revealed the presence of amphetamine in the demyelinated area, which might be considered as the pathogenic agent, since other causes for demyelination could be excluded. This case represents the first report showing a demyelinating process due to a street drug.

## 1. Introduction

Amphetamines are sympathomimetic drugs with a central stimulant activity. Clinical manifestations of toxicity are similar to those of cocaine and include sudden death due to cardiac arrhythmia, stroke, seizures, psychosis, and rhabdomyolysis [1]. Neuropathologic alterations of amphetamine abuse include cerebral infarcts and hemorrhage, often caused by emboli associated with cardiac arrhythmias or myocardial infarction [2]. Furthermore, vasospasm or vasculitis due to the pharmacologic action of amphetamines may be causative factors [1, 2]. We report a patient with an acute demyelination associated with amphetamine abuse which has not been previously described.

## 2. Case Report

A 31-year-old woman was admitted to a hospital due to chest pain. A tentative diagnosis of anorexia nervosa was rendered, as she reported weight loss of 60 kg within a few months. The next day she developed cough, fever, and leukocytosis subsequently manifested dyspnea, cyanosis, and hypotension, and eventually required catecholamines. Despite assisted breathing, the lung and heart function deteriorated progressively. Furthermore, she developed acute renal and hepatic failure. Despite antibiotic therapy, she developed progressive bronchopleural fistulas and a therapy-resistant *Burgholderia cepacia* pneumonia. The patient developed signs of brain edema (anisocoria, dilated pupils) and finally died due to multi-organ failure. Blood testing for HIV-1 antibodies was negative. There was no clinical evidence of multiple sclerosis.

**Figure 1:**
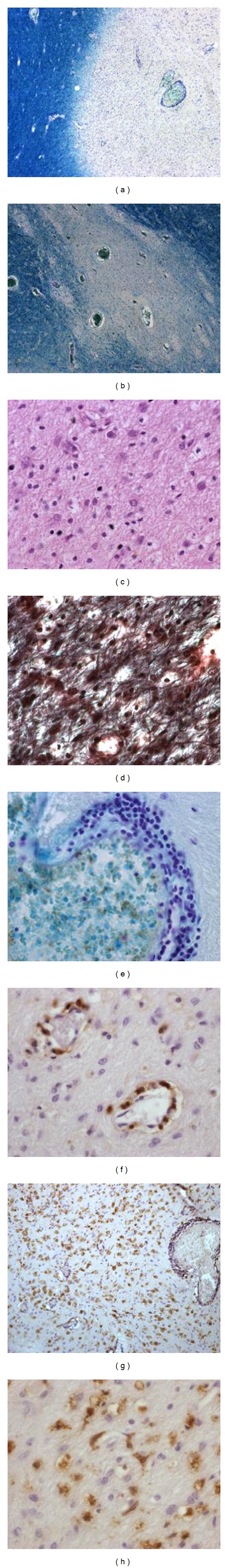
Microphotographs of the lesion in the occipital lobe showing a sharply demarcated area of demyelination ((a) and (b) Luxol-Fast-Blue, magnification 4X), reactive astrogliosis ((c), (h), and (e) stain, magnification 100X), and unaffected nerve fibers ((d) Bodian stain, magnification 200X). Note the perivascular lymphocytic infiltrates ((e) Luxol-Fast-Blue stain, magnification 100X), perivascular T-lymphocytic infiltrates and lymphocytes in the surrounding parenchyma ((f) UHCL antibody, magnification 200X), and activated macrophages/microglia within the demyelinated area (CR3/43 antibody; (g) magnification 25X, (h) magnification 400X).

**Figure 2:**
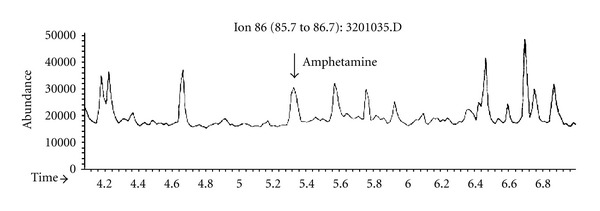
Gas chromatography-mass spectrometry (GCMS) of the brain tissue: single-ion monitoring for amphetamine (target ion 86 dalton, retention time 5.33 mins). Calibration was performed with spiked serum samples up to 0.1 mg/L and led to a concentration of 0.017 *μ*g/g brain tissue.

The general autopsy revealed severe bronchopneumonia and myocarditis. The brain weighed 1,250 grams. Besides signs of moderate brain edema, the macroscopic examination was unremarkable. Microscopic examination demonstrated moderate hypoxic neuronal damage, nerve cell loss, and moderate edematous changes. In the white matter of the occipital lobe, two sharply demarcated areas of demyelination were evident (Figures 1(a) and 1(b)) associated with reactive astrogliosis (Figure 1(c)). The axons were well preserved on Bodian silver impregnation (Figure 1(d)). There was a diffuse and perivascular infiltration of T-lymphocytes as seen on immunostaining with UCHL (CD45RO) antibody (Figure 1(e)) as well as numerous CD68- and CR3/43-immunopositive macrophages/microglial cells (Figures 1(g) and 1(h)). The cerebellum and brain stem were unremarkable. Immunohistochemical investigations for the presence of infectious agents (HIV-1, VZV, CMV, and HSV-1, *Toxoplasma gondii*) were negative. Toxicological analysis with gas chromatography (GC) and mass spectrometry (MS) of a white powder which was found in the bag of the patient on admission revealed an amphetamine content of 14.54%. Analysis of brain samples of the occipital region including the demyelinated area revealed an amphetamine content of 0.017 *μ*g/g determined by GC/MS (Figure 2). Examined samples of brain tissue adjacent to the lesion of the same and of other brain slabs did not reveal any evidence for amphetamine. Although this is a very low level, which was determined after 6 weeks in the hospital, it indicates that a high blood level of amphetamine would have been possible before hospital admission. However, on admission, the clinicians never had a suspicion of drug abuse, and therefore initial drug screening on blood or urine has not been performed.

## 3. Discussion

Amphetamines are sympathomimetic drugs with a central stimulant activity. Clinical manifestations of toxicity are similar to those of cocaine and include sudden death due to cardiac arrhythmia, stroke, seizures, psychosis, and rhabdomyolysis [1]. Amphetamines are anorectic drugs by virtue of their ability to cause the release and/or prevent the reuptake of noradrenaline and dopamine [1]. Therefore, the rapid and extensive weight loss within a few months, as seen in our patient, might be due to a long-lasting amphetamine abuse.

Neuropathologic alterations of amphetamine abuse include cerebral infarcts and hemorrhage, often caused by emboli associated with cardiac arrhythmias or myocardial infarction [2]. So far, to our knowledge, acute demyelination has not yet been described in cases of amphetamine abuse. Disorders affecting the white matter can be classified into inherited metabolic disorders, that is, the leukodystrophies (including metachromatic leukodystrophy, Krabbe disease, adrenoleukodystrophy, Alexander disease, and Pelizaeus-Merzbacher disease) and demyelinating disorders [3]. Based on their most probable cause, the demyelinating disorders may be classified as immune-mediated (multiple sclerosis), viral (progressive multifocal leukoencephalopathy) [4], toxic (central pontine myelinolysis, Marchiafava-Bignami disease) or chronic progressive subcortical demyelination (Binswanger disease) and leuko-araiosis as well as ischemic [5]. In general, demyelination may be induced by serum factors or degradative cell products secreted by activated macrophages, which, in turn, may be stimulated by products of activated lymphocytes [6, 7]. The process of demyelination is usually preceded by an increased permeability of the blood-brain barrier. In our case, we could exclude most of the common viruses, toxic agents like alcohol, as well as ischemia and hypoxia. Multiple sclerosis has to be considered in the differential diagnosis of this case. Thus, the two demyelinating foci found in the occipital lobe might have been incidental findings representing the early phase of MS which went undiagnosed. Since their size is quite small, they might not have been able to produce clinical features related to the specific brain region (most probably visual dysfunctions). These functional disturbances could have been present but not noted as the patient was abusing drugs. However, the presence of amphetamine within the demyelinating lesions makes it less likely that MS is the cause. As a reverse conclusion, one would have to find also amphetamine traces in demyelinating lesions in cases clinically diagnosed as having MS in order to diagnose the present case as MS. Furthermore, amphetamine was only detected in the demyelinating foci, but not in adjacent or far distant brain regions. Based on the lack of other evidence, we conclude that the lesion is most probably due to the direct action of amphetamine. Data supporting this hypothesis are given by the observation that inhalation of preheated heroin causes a spongiform leukoencephalopathy with marked neurological deficits noted several weeks after exposure [8, 9]. CT and MRI findings usually demonstrate myelin damage of the white matter of the cerebral hemispheres and cerebellum [9]. The pathogenesis of the observed leukoencephalopathy in these cases is still unclear. A lipophilic toxin-induced process was considered to be due to contaminants and induced or enhanced by cerebral hypoxia, but a definite toxin could not be identified [8, 9]. Further support is given by the fact that the amphetamine derivatives 3,4-methylenedioxymethamphetamine (MDMA) and 3,4-methylenedioxyamphetamine (MDA) were demonstrated by immunohistochemistry in all cortical brain regions and the neurons of the basal ganglia, the hypothalamus, the hippocampus, and the cerebellar vermis of two amphetamine abuse fatalities [10]. Immunoreactivity was also seen in the white matter [10]. One gene expression analysis of amphetamine administration in rats showed significantly altered gene expression for genes related to transcription factors, cellular stress/molecular chaperones, signalling pathways, synaptic function, protein synthesis/degradation, and others [11]. Unfortunately, no white matter regions were sampled. However, it was recently shown by microarray studies that the chronic abuse of cocaine in humans results in a significant decrease in the expression of myelin-related genes including myelin basic protein (MBP), proteolipid protein (PLP), and myelin-associated oligodendrocyte basic protein (MOBP) [12]. In vivo administration of amphetamine resulted in a dose-related enhancement of neurological and histological signs of acute experimental allergic encephalomyelitis [13]. Furthermore, widespread axonal damage with concomitant microglia activation has been shown in the brains of polydrug abusers [14]. It is noteworthy that, for the multitude of animal experiments done so far, damage to the white matter was never reported. Given the fact that the volume fraction of white matter in the mouse or rat brain is significantly lower than that in the human brain, it might be concluded that myelin is less vulnerable in these animals and, thus, no changes could be seen; or possible white matter changes were never examined. In this context the use of amphetamine as a treatment for multiple sclerosis [14] should be seen with caution.

This is the first report in which the demonstration of amphetamine in the affected brain region strongly suggests a direct link between amphetamine abuse and demyelination; however, the pathogenetic mechanisms of the demyelination still remain unclear.

## References

[1] Karch S (2002). *Karch’s Pathology of Drug Abuse*.

[2] Delaney P, Estes M (1980). Intracranial hemorrhage with amphetamine abuse. *Neurology*.

[3] Suzuki K, Garcia JH (1997). Demyelinating disorders and leukodystrophies. *Neuropathology. The Diagnostic Approach*.

[4] Johnson RT, Major EO, Lazzarini RA (2004). Infectious demyelinating diseases. *Myelin Biology and Disorders*.

[5] Stys PK, Waxman SG, Lazzarini RA (2004). Ischemic white matter damage. *Myelin Biology and Disorders*.

[6] Cuzner ML, Norton WT (1996). Biochemistry of demyelination. *Brain Pathology*.

[7] Noseworthy JH (1999). Progress in determining the causes and treatment of multiple sclerosis. *Nature*.

[8] Schiffer D, Brignolio F, Giordana MT, Mongini T, Migheli A, Palmucci L (1985). Spongiform encephalopathy in addicts inhaling pre-heated heroin. *Clinical Neuropathology*.

[9] Wolters EC, Stam FC, Lousberg RJ (1982). Leucoencephalopathy after inhaling “heroin“ pyrolysate. *The Lancet*.

[10] De Letter EA, Espeel MFA, Craeymeersch MEC (2003). Immunohistochemical demonstration of the amphetamine derivatives 3,4-methylenedioxymethamphetamine (MDMA) and 3,4-methylenedioxyamphetamine (MDA) in human post-mortem brain tissue and the pituitary gland. *International Journal of Legal Medicine*.

[11] Sokolov BP, Polesskaya OO, Uhl GR (2003). Mouse brain gene expression changes after acute and chronic amphetamine. *Journal of Neurochemistry*.

[12] Albertson DN, Pruetz B, Schmidt CJ, Kuhn DM, Kapatos G, Bannon MJ (2004). Gene expression profile of the nucleus accumbens of human cocaine abusers: evidence for dysregulation of myelin. *Journal of Neurochemistry*.

[13] Núñez MJ, Balboa J, Rey-Méndez M (2007). Effects of amphetamine and cocaine on the development of acute experimental allergic encephalomyelitis in Lewis rats. *Human and Experimental Toxicology*.

[14] Benedict RHB, Munschauer F, Zarevics P (2008). Effects of l-amphetamine sulfate on cognitive function in multiple sclerosis patients. *Journal of Neurology*.

